# Evaluation of Antibacterial Properties of Triorganotin Carboxylates Containing Functionalised Ester Groups in Tests Against Some Pathogenic Bacteria

**DOI:** 10.1155/MBD.2001.107

**Published:** 2001

**Authors:** J. Koshy, A. Ansary, K. M. Lo, V. G. Kumar Das

**Affiliations:** 1 Institute for Postgraduate Studies and Research University of Malaya Kuala Lumpur 50603 Malaysia; 2 Institute of Biological Sciences University of Malaya Kuala Lumpur 50603 Malaysia; 3 Department of Chemistry University of Malaya Kuala Lumpur 50603 Malaysia

## Abstract

Bacterial screening employing the agar diffusion test on triphenyltin carboxylates containing various functional residues in the ester moiety revealed appreciable differences in their activities relative to triphenyltin acetate. Among these, [3-(Diethylphosphono)propionato] triphenyltin (**1**)  and [*N*-cyclohexylcarbamoyl) glycinato] triphenyltin displayed activities comparable to tri-*n*-butyltin cinnamate (**2**) towards both Gram-positive and Gram-negative bacteria; the latter compound was the most active among the eleven triorganotin compounds tested, which included cyclopentyldiphenyltin hydroxide (**3**) and its methacrylate derivative. Applying the more quantitative plate count and optical density tests on compounds **1-3**, it was shown that their inhibitory activity ranked in the order **2 > 3 >1**. Significantly, **3** caused around 90% inhibition of both *Eschechia coli *(−) and *Pseudomonas aeruginosa* (−) when incubated for 24 h at 37±1℃ at the 10.0 μg/ mL concentration level. Compound **2** was less effective against *P.aeruginosa* than against *E.coli*. While the Gram-positive bacteria were all readily inhibited, *Bacillus subtilis* (+) appeared to the most susceptible among them towards the test compounds.


### EVALUATION OF ANTIBACTERIAL PROPERTIES OF TRIORGANOTIN CARBOXYLATES CONTAINING FUNCTIONALISED ESTER GROUPS IN TESTS AGAINST SOME PATHOGENIC BACTERIA

J. Koshy[1], A. Ansary[2], K. M. Lo[3] and V. G. Kumar Das[*3]

[1] Institute for Postgraduate Studies and Research, [2] Institute of Biological Sciences,
[3] Department of Chemistry,
University of Malaya, 50603 Kuala Lumpur, Malaysia

**Abstract**

Bacterial screening employing the agar diffusion test on triphenyltin carboxylates containing various functional residues in the ester moiety revealed appreciable differences in their activities relative to triphenyltin acetate. Among these, [3-(Diethylphosphono)propionato] triphenyltin (**1**) and [*N*-cyclohexylcarbamoyl) glycinato] triphenyltin displayed activities comparable to tri-*n*-butyltin cinnamate (**2**) towards both Gram-positive and Gram-negative bacteria; the latter compound was the most active among the eleven triorganotin compounds tested, which included cyclopentyldiphenyltin hydroxide (**3**) and its methacrylate derivative. Applying the more quantitative plate count and optical density tests on compounds **1-3**, it was shown that their inhibitory activity ranked in the order **2** > **3** >**1**. Significantly, **3** caused around 90% inhibition of both *Escherichia coli* (-) and *Pseudomonas aeruginosa* (-) when incubated for 24 h at 37±1°C at the 10.0 μg/ mL concentration level. Compound **2** was less effective against *P.aeruginosa* than against *E.coli*. While the Gram-positive bacteria were all readily inhibited, *Bacillus subtilis* (+) appeared to the most susceptible among them towards the test compounds.

### Introduction

In previous reports on the enhanced biological properties manifested by new organotin(IV) derivatives, we have elaborated on their antifungal[1,2] and insecticidal[3-6] activities and also presented our results on the evaluation of their genotoxic potential[7]. In additional reports on such compounds we have discussed their interesting anti-tumour properties[8,9] shown in *in-vitro* screening tests against a range of human cancer cell lines. In this paper, we report the antibacterial effects of triphenyltin cinnamates and other functionalised esters along with tributyltin cinnamate and two diphenylcyclopentyltin compounds in tests against three Gram-positive and two Gram-negative species of pathogenic bacteria. Our study was prompted by the general observation presented in the literature that most organotin compounds, including tri-*n*-butyltin benzoate and bis(tri-*n*-butyltin) oxide which already find application as major active ingredients in some commercial disinfectants, are significantly more active against Gram-positive bacteria than against Gram-negative bacteria.[10-13] The exceptions are tri-*n*-propyltins and certain diorganotin compounds which are relatively more active against Gram-negative bacteria,[14] but weighted against the application of the tri-*n*-propyltins is their relatively high mammalian toxicity compared to tri-*n*-butyltin and triphenyltin compounds.[15,16] The work reported herein was initiated with the aim of obtaining an organotin compound with two or three phenyl groups on tin that would be effective as a bacteriostat against both Gram-positive and Gram-negative bacteria.

### Materials and Methods

Except for two new organotin compounds whose synthesis is described below, the remainder were prepared as previously reported.[18-20] Solutions of the compounds in absolute ethanol corresponding to concentrations of 0.1, 2.5, 5.0, 7.5 and 10.0 μg / mL were prepared from a 400 μg / mL stock solution, and were used in the Agar Diffusion test for determining the Minimum Inhibitory Concentration (MIC) against the test bacteria . The organotin concentration range 2.5, 5.0, 7.5, 10.0 and 25.0 μg/ mL was employed in additional (more quantitative) tests based on plate count and optical density measurements. All the solutions were sterilised by passage through 0.2μ nitrocellulose membrane filters (Millipore). The five species of bacteria, *Bacillus subtilis* (+), *Staphylococcus aureus* (+), *Streptococcus pyogenes* (+), *Escherichia coli* (-) *and Pseudomonas aeruginosa* (-), used in this work were obtained from the Microbiology Culture Collection of the Institute of Biological Sciences, University of Malaya. Nutrient agar or nutrient broth (Oxoid) were used for the growth of the bacteria, except in the case of *Streptococcus pyogenes* where Brain Heart Infusion (BHI) agar or broth were used as the growth medium.

***Synthesis of [N-cyclohexylcarbamoyl) glycinato] triphenyltin***

This was prepared by the condensation reaction of triphenyltin hydroxide with cyclohexylhydantoic acid, $C_6H_{11}NHCONHCH_2COOH$. The latter was prepared as follows:
10 g of glycine and 7 g of sodium hydroxide were dissolved in 200 mL of water. To this was added 22.3 mL of cyclohexylisocyanate and the reaction mixture was stirred vigorously for 6 h. The insoluble bis(cyclohexyl)urea obtained as a by-product was filtered off, and the filtrate was acidified with dilute hydrochloric acid. The white solid formed was filtered, washed with water and re-crystallised from dilute ethanol to give 14.7 g of cyclohexylhydantoic acid, m.p.171-173 °C.
The triphenylstannyl ester was prepared by heating briefly 3.71g of the above acid with 6.63 g of triphenyltin hydroxide in ethanol and allowing the solution to cool. The product was a white solid, m.p. 190-192 °C. Analysis: Found: C, 58.5; H, 5.11; N, 4.76. $C_{27}H_{30}O_3N_2Sn$ Calcd.: C, 58.9; H, 5.45; N, 5.09 %.

***Synthesis of Cyclopentyldiphenyltin methacrylate***

This was prepared by mixing ethanolic solutions of equimolar quantities of cyclopentyldiphenyltin hydroxide and methacrylic acid and briefly warming the mixture on a water bath. A white solid was formed with a decomposition point exceeding 330 °C. The product was characterised spectroscopically.
$^1H$ *NMR* (DMSO-$d_6$ / $CDCl_3$ ): δ (ppm, rel. to TMS) 1.23-2.60 (m, *cyclo*-$C_5H_9$ ) ; 7.11-7.66 (m, Ph );
5.95 (d, =$CH_2$); 1.68 (s, Me). IR *(Nujol) : (carboxyl str.),* 1698.9 *cm*$^{-1}$ *; hydroxyl str., absent.*

***Agar Diffusion test***

Sterilised paper discs of Whatman No.1 filter paper with a diameter of 12 mm were soaked in the organotin test solutions of varying concentrations. The discs were then aseptically placed on nutrient (or BHI) agar medium in Petri dishes which had been previously inoculated with the respective bacteria and incubated at 37±1°C. A bacterial count of $10^4$ was ensured in each of the Petri dishes prior to the commencement of the test. The inhibition zone that was formed around each disc containing the organotin test compound was measured (in mm) after 24 h; the incubation period was extended to 48 h in the case of *S. pyogenes* . The control contained only the filter paper disc soaked in ethanol. The effect of each organotin concentration was investigated in duplicate. For this purpose, it proved expedient to position five filter paper discs, soaked respectively in the solutions of the five organotin concentrations, in separate segments of the Petri dish for each experiment involving a given test compound. The concentration level of organotin that yields a zone of inhibition whose diameter minimally exceeds that shown by the ethanol control was taken as the MIC value.

***Plate Count and Optical Density tests***

The bacterial inoculum used for these tests was a 24 h culture in 3 mL of nutrient broth or BHI broth as appropriate. This was added to 300 mL of the relevant broth in a flask and incubated at 37±1°C on a shaker incubator operating at 250 rpm and shaken for a 24 h period. Two more cultures were similarly duplicated in a second and a third flask, respectively. Samples were withdrawn aseptically at one-hour intervals in order to estimate the growth rate. This was done by measurements of the optical density of the bacteria at 550 nm as well as by plate count ("viable count") based on serial dilution. The log-phase growth of the bacteria was achieved about 4 h after the introduction of the inoculum, at which point the organotin solution of known concentration was added (flask 1). The same volume of ethanol was added to flask 2. The ethanol sensitivity was checked using flask 3, which contained no additives, as control. In general, only at high organotin concentrations (corresponding to larger volumes of the stock solution added to the broth) was some difference noted between measurements made using aliquots from flasks 2 and 3 for some bacteria, necessitating corrections to be made for the ethanol sensitivity. This was particularly the case with the Gram-negative bacteria whose cell walls are known to be more lipophilic. For the optical density (OD) measurements , 1 mL aliquots were withdrawn hourly from the flasks. Inhibition of the bacteria by the organotin resulted in a drop in optical density. The percentage inhibition was determined using the formula,

$$\% \text{ Inhibition} = [\, OD_{(control)} - OD_{(organotin)} \,] \,/\, [\, OD_{(control)} \,] \times 100.$$

For the plate count, 0.1 mL of the sample was withdrawn and this was diluted with 0.9 mL of 0.85% saline solution. A serial dilution (3x) using the same proportions was next performed; 0.1 mL of the last two dilutions (i.e. corresponding, respectively, to dilution factors of $10^3$ and $10^4$) were plated out and spread on nutrient (or BHI) agar plates and incubated at 37±1°C for 24 h. The number of colonies which appeared on the agar plates was taken as the measure of the number of viable (surviving) cells. Each colony was regarded as a single Colony Forming Unit (CFU). Thus CFU/mL (viable count) = Number of colonies x Dilution factor x 10. By way of illustration, the following data are presented for the case of the inhibition of *Staphylococcus aureus* (+) by tri-*n*-butyltin cinnamate at the 2.5 μg/ mL concentration level added to log-phase cells of the bacterial broth at the $5^{th}$ hour.

| Time | 0 h | 2 h | 4 h | 5 h | 6 h | 7 h | 8 h |
|---|---|---|---|---|---|---|---|
| Viable count (flask 1) CFU/mL | 8x10$^5$ | 10x10$^5$ | 4x10$^6$ | 6x10$^6$ | 2x10$^6$ | 1x10$^6$ | 1x10$^6$ |
| Viable count (flask 2) CFU/mL | 4x10$^5$ | 10x10$^5$ | 1x10$^6$ | 3x10$^7$ | 2x10$^7$ | 1x10$^8$ | 6x10$^8$ |

Based on the above data, % inhibition $= \frac{(6 \times 10^6 - 1 \times 10^6)}{6 \times 10^6} \times 100 = 83$

A comparable result was obtained using optical density data.

| Treatment | Optical Density | | | | | | |
|---|---|---|---|---|---|---|---|
| | 0 h | 2 h | 4 h | 5 h | 6 h | 8 h | 9 h |
| Bacterium + organotin | 0.01 | 0.02 | 0.20 | 0.25 | 0.30 | 0.20 | 0.2 |
| Bacterium + ethanol | 0.02 | 0.02 | 0.14 | 0.30 | 0.50 | 1.0 | 1.2 |

Thus: % Inhibition $= \frac{(1.0 - 0.20)}{1.0} \times 100 = 80$

**Results and Discussion**

The results of the Agar Diffusion test are presented in Table 1. Based on a consideration of the MIC values and the corresponding diameters of the inhibition zones, it is seen that tri-*n*-butyltin cinnamate was the

Table 1: Minimum Inhibitory Concentrations and Diameters of inhibition zones (values in italics) for organotins tested against pathogenic bacteria

| **Organotin compound** | **MIC (μg/ mL)** ***Diameter of inhibition zone( mm)**** | | | | |
|---|---|---|---|---|---|
| | *Bacillus subtilis*(+) | *Staph. aureus*( +) | *Strept. pyogenes*(+) | *Esch. coli*(-) | *Pseud. aerug.* (-) |
| $Ph_3SnOC(O)CH: CHC_6H_5$ | 0.1<br>*1.5* | 0.1<br>*2.5* | 5.0<br>*2.0* | 5.0<br>*1.0* | 2.5<br>*1.0* |
| $Ph_3SnOC(O)CH: CHC_6H_4$-4 $NO_2$ | 7.5<br>*1.5* | 0.1<br>*1.5* | 0.1<br>*2.25* | 0.1<br>*2.0* | 2.5<br>*2.0* |
| $Ph_3SnOC(O)CH: CHC_6H_4$ –4 Me | 7.5<br>*2.5* | 0.1<br>*3.0* | 5.0<br>*1.5* | 2.5<br>*2.0* | 2.5<br>*1.0* |
| $Ph_3SnOC(O)CH_2CH_2P(O)(OEt)_2$ | 2.5<br>*2.0* | 5.0<br>*2.5* | 0.1<br>*3.0* | 2.5<br>*2.0* | 0.1<br>*3.5* |
| $Ph_3SnOC(O)CH_2NHC(O)NH$(*cy*-$C_6H_{11}$) | 2.5<br>*3.0* | 0.1<br>*2.0* | 2.5<br>*2.0* | 0.1<br>*4.0* | 2.5<br>*3.0* |
| $[Ph_3SnOC(O)C_6H_4$-2 $SO_3]^-$ [ *cy*-$C_6H_{11})_2NH_2]^+$ | 2.5<br>*2.0* | 5.0<br>*1.5* | 5.0<br>*3.0* | 5.0<br>*1.0* | 2.5<br>*2.5* |
| $Ph_3SnOC(O)CH_3$ | 2.5<br>*4.0* | 7.5<br>*1.5* | 2.5<br>*3.25* | 0.1<br>*2.0* | 2.5<br>*2.0* |
| $Ph_3SnOH$ | 2.5<br>*3.75* | 2.5<br>*2.0* | 2.5<br>*3.5* | 2.5<br>*2.5* | 5.0<br>*2.25* |
| $Ph_2$(*cy*-$C_5H_9$)SnOH | 2.5<br>*1.0* | 5.0<br>*1.0* | 2.5<br>*2.0* | 10.0<br>*3.0* | 5.0<br>*3.0* |
| $Ph_2$(*cy*-$C_5H_9$)SnOC(O)C(Me):$CH_2$ | 5.0<br>*2.0* | 2.5<br>*1.5* | 0.1<br>*2.0* | 5.0<br>*1.0* | 5.0<br>*2.0* |
| *n*-$Bu_3SnOC(O)CH:CHC_6H_5$ | 0.1<br>*12* | 2.5<br>*2.0* | 2.5<br>*2.0* | 0.1<br>*8* | 0.1<br>*1.0* |

*corrected for inhibition by ethanol where this was noticeable, particularly for the Gram-negative bacteria most active among the compounds tested against all the pathogens, and especially so against *Bacillus subtilis* (+) and *Escherichia coli* (-). The triphenyltin cinnamates revealed some differences in activity with the nature of the *para*-substituent present on the esteryl phenyl group. While the unsubstituted triphenyltin cinnamate was active against *Bacillus subtilis* (+) at the low MIC value of 0.1 µg/ mL, both the *para*-methyl and *para*-nitro derivatives were less effective. Overall, however, the *para*-nitro compound registered the highest activity among the triphenyltin cinnamates, and was also more active than the commercial crop protectants,[21] triphenyltin acetate and –hydroxide.

The effect of varying esteryl functionalities in the triphenyltin series was more strikingly revealed with *catena-O,O'*-[3-(Diethylphosphono)propionato] triphenyltin and [*N*-cyclohexylcarbamoylglycinato] triphenyltin, both of which showed activities comparable to tributyltin cinnamate. Particularly noteworthy was their high activity towards the gram-negative bacteria. At the 0.1 µg/mL MIC value, the cyclohexylcarbamoylglycinato compound was particularly effective against *E.coli*, while the phosphonopropionate was the most active among the compounds tested against *P.aeruginosa*, a pathogen that exhibits resistance to a wide variety of antimicrobial drugs.[22] The stannate ester, Dicyclohexylammonium *catena*-(2-sulfobenzoate-*O,O'*)-triphenylstannate(IV), was the least active among the triphenyltin compounds. Its activity was comparable to cyclopentyldiphenyltin hydroxide and –methacrylate. These observations suggest that variations in the anionic residues on tin can bring about marked differences in bacterial activity similar to those encountered with variations in the tin-carbon skeletal groups - alkyl, cycloalkyl or aryl.

To investigate more quantitatively the antibacterial effects,[3-(Diethylphosphono)propionato] triphenyltin (**1**), tri-*n*-butyltin cinnamate (**2**), and cyclopentyldiphenyltin hydroxide (**3**) were selected for evaluation by the more sensitive plate count and/or optical density measurements, the results of which are presented in Table 2. The data, while reinforcing the stronger antibacterial properties of **2,** also allowed of some interesting inferences. Compound **3** emerged to be generally more effective than **1** against the range of bacteria studied. Indeed, at the same concentration level of 10.0 µg/ mL and for incubation periods of organotin with the bacteria extended to 18-20 hours, the heteroleptic triorganotin compound **3** proved to be appreciably more effective than the homoleptic compound **1** against both the Gram-negative bacteria, registering around 90% inhibition. The negligible inhibition of *P.aeruginosa* (-) by **2** at the 7.5µg/ mL concentration level attests to the relatively more resistant nature of this bacterium. Conceivably, its inhibition could have been achieved either with longer periods of incubation or higher organotin concentration. While the Gram-positive bacteria were all readily inhibited, *B.subtilis* appeared to the most susceptible among them towards the test compounds.

Table 2: Evaluation of antibacterial properties of [3-(Diethyl-phosphono)propionato] triphenyltin (**1**), tri-*n*-butyltin cinnamate (**2**) and cyclopentyldiphenyltin hydroxide (**3**) by the plate count and optical density methods

| Bacteria | Method[a] | % Inhibition (I) I (conc.)[b] / time[c] | | |
|---|---|---|---|---|
| | | **1** | **2** | **3** |
| *B.subtilis* (+) | A | 89 (5.0) / 6 | 100 (2.5) / 8 | 100 (5.0) /24 |
| | B | | 96 (2.5) / 9 | |
| *S.aureus* (+) | A | | 83 (2.5) / 8 | |
| | B | 88 (25.0) / 24 | 89 (2.5) / 9 | 89 (7.5) / 24 |
| *S.pyogenes* (+) | A | | 90 (5.0) / 8 | |
| | B | 50 (5.0) / 24 | 94 (5.0) / 9 | 67 (5.0) / 24 |
| | B | 75 (10.0) / 24 | | |
| *E.coli* (-) | A | 60 (25.0) / 24 | 78 (7.5) / 9 | 96 (10.0) / 24 |
| *P.aeruginosa* (-) | A | 65 (10.0) / 24 | 0 (7.5) / 9 | 88 (10.0) / 24 |
| | B | 67 (10.0) / 24 | | |

[a] A (plate count), B (optical density); [b] concentration (µg/ mL) of organotin added to broth at log-phase growth of bacteria ; [c] time interval (h) following initial bacterial inoculation of the broth at which the values of the viable count and/or optical density were taken for calculating the percentage inhibition.

**Acknowledgements**

This work was supported by the National Science Council for Research and Development (Grant No.2-07-04-06), with additional support provided by Organotin (M) Sdn. Bhd.

**References**

1. A.J.Kuthubutheen, R.Wickneswari and V.G.Kumar Das, *Appl. Organomet.Chem.*, **3**(1989)231; *ibid.*, **3** (1989) 243; *ibid.*, **3** (1989) 309; *ibid.*, **3** (1989) 319; *ibid.*, **3** (1989) 451.
2. A.J.Kuthubutheen, Y.Salahudin and V.G.Kumar Das, in: *"Chemistry and Technology of Silicon and Tin"* (Eds: V.G.Kumar Das,S.W.Ng and M.Gielen), Oxford Sc.Publ., Oxford, 1992, Chapter 20, pp.289.
3. Nazni W.Ahmad, Sofian-Azirun Mohd., S.Balabaskaran and V.G.Kumar Das, *Applied Organomet.Chem.*,**7**(1993)583.
4. Nazni W.Ahmad, Tay Siew Huang, S.Balabaskaran, K.M.Lo and V.G.Kumar Das, *Met.-based Drugs,* **1** (1994) 1.
5. S. Selvaratnam, S.W.Ng, Nazni W.Ahmad and V.G.Kumar Das, *Main Group Met. Chem.*, **5** (1999) 321.
6. V.G.Kumar Das. S.W.Ng and K.M.Lo, in: *"Main Group Elements and their Compounds"* (Ed. V.G.Kumar Das), Narosa Publ.House/Springer Verlag, 1996, pp.369-386.
7. Sam Choon Kook, M.K.Ng and V.G.Kumar Das, *Applied Organomet.Chem.*,**5** (1991) 409.
8. S.W.Ng, V.G.Kumar Das and M.Gielen, *Appl.Organomet.Chem.*,**6** (1992) 489; S.W.Ng, V.G.Kumar Das, M.Gielen and E.R.T.Tiekink, *ibid,* **6** (1992) 19; S.W.Ng, V.G.Kumar Das, J.Holecek, A.Lyeka, M.Gielen and M.G.B.Drew, *ibid,* **11** (1997) 39.
9. J.Koshy, V.G.Kumar Das, S.Balabaskaran, S.W.Ng and Norhanom Wahab, *Met.-based Drugs*,**7** (2000) 245.
10. C.J.Evans and S.Karpel, in: *Organotin Compounds in Modern Technology*, Elsevier, Amsterdam,1985, pp.232-248.
11. R.C.Poller, in: *"The Chemistry of Organotin Compounds"*, Chapter 14 (and references therein), Logos, London, 1970.
12. T.N.Srivastava, A.K.Sengupta and S.P.Jain, *Ind.J.Chem. Sect.A*, **21A** (1982) 384, and references therein.
13. Talal A.K.Al-Allaf, Redha H.Al-Bayati and Subhi H.Khalaf, *Appl. Organomet.Chem.*, **7** (1993) 635.
14. A.G.Davies and P.J.Smith, in: *"Comprehensive Organometallic Chemistry"* (Eds. G. Wilkinson, F.G.A.Stone and E.W.Abel), Vol.2, Pergamon Press, Oxford, 1982, pp.519-627.
15. P.J.Smith and V.G.Kumar Das, in: *"Main Group Elements and their Compounds"* (Ed. V.G.Kumar Das), Narosa Publ.House/Springer Verlag, 1996, pp.398-411.
16. J.G.A.Luijten and O.R.Klimmer, in: *"Toxicological Data on Organotin Compounds"* (Ed. P.J.Smith), ITRI Publ. No.538, 1978, International Tin Research Institute, Uxbridge, London; F.A.O. and W.H.O., Rome, 1971, AEP: 1979/M/12/1, 327-366.
17. S.W.Ng and V.G.Kumar Das, *J.Cryst.and Spectros.Res.*,**22** (1992) 507.
18. Ong Ghee Chee. K.M.Lo and V.G.Kumar Das, *Main Group Met. Chem.*, **16** (1993) 101.
19. Siah Lay Foong, S.W.Ng, M.Gielen and V.G.Kumar Das, *Malaysian.J. Sci.*, **15B** (1994) 13.
20. S.W.Ng and V.G.Kumar Das, *Acta Cryst.*, **C52** (1996) 1373.
21. R.Bock, in: *Residue Reviews*, vol. 79 (Ed. F.A.Gunther), Springer-Verlag, 1981.
22. R.Cruickshank, T.P.Duguid, B.P.Marmion and R.H.A.Swain, in: *Medical Microbiology*, vol II, Churchill Livingstone, Edinburgh, 1975.

**Received: March 26, 2001 – Accepted: May 3, 2001 – Accepted in publishable format: May 14, 2001**

